# Aquatic Plant Genomics: Advances, Applications, and Prospects

**DOI:** 10.1155/2017/6347874

**Published:** 2017-08-16

**Authors:** Shiqi Hu, Gaojie Li, Jingjing Yang, Hongwei Hou

**Affiliations:** ^1^The State Key Laboratory of Freshwater Ecology and Biotechnology, The Key Laboratory of Aquatic Biodiversity and Conservation of Chinese Academy of Sciences, Institute of Hydrobiology, Chinese Academy of Sciences, Wuhan, Hubei 430072, China; ^2^University of Chinese Academy of Sciences, Beijing 100049, China

## Abstract

Genomics is a discipline in genetics that studies the genome composition of organisms and the precise structure of genes and their expression and regulation. Genomics research has resolved many problems where other biological methods have failed. Here, we summarize advances in aquatic plant genomics with a focus on molecular markers, the genes related to photosynthesis and stress tolerance, comparative study of genomes and genome/transcriptome sequencing technology.

## 1. Introduction

Since the idea was founded in 1986, genomics has become one of the most active and influential leading-edge areas of life sciences. Genome annotation and functional genomics have developed rapidly and penetrated into many areas of life science, profoundly affecting the future development and direction of research. Since the complete genome sequence was demonstrated for the model plant Arabidopsis (*Arabidopsis thaliana*) [1] and subsequently for rice (*Oryza sativa*) [2], whole-genome sequencing (WGS) of many species of plants has been carried out [3–6]. However, only a few aquatic plants are among them.

Aquatic plants play an important role in water purification and landscaping. They are also a source of bioenergy, biomass, and human/animal food. The study of aquatic plants, especially at molecular levels, has been of increasing interest. In this review, progress in aquatic plant genomics research involving development and application of molecular markers, comparative genomics, functional genomics, and genome sequencing, as well as future prospects, is summarized.

## 2. Molecular Marker: Development and Application in Genomics Research

DNA-based molecular markers are the most powerful diagnostic tools used to detect genetic polymorphisms at the level of DNA. Currently, dozens of different molecular markers have been employed to assess genetic diversity for phylogenetic analysis and to identify germplasm.

### 2.1. Genetic Diversity Analysis

Aquatic plants are rich and varied worldwide, including 87 families, 168 genera, and 1022 species [7], resulting in complex and genetically diverse ecosystems. Scientists use molecular markers such as SSR (simple sequence repeat), RAPD (random amplified polymorphic DNA), RFLP (restriction fragment length polymorphism), AFLP (amplified fragment length polymorphism), and ISSR (Intersimple sequence repeat) to reveal individual or group differences among aquatic plants. SSR, also known as microsatellite, is most widely used in the study of genetic diversity of aquatic plants due to its high abundance, high variability, and codominance. For example, SSR analysis of an aquatic macrophyte *Sparganium emersum* revealed significant genotypic diversity between populations in two rivers, the Swalm and Rur. These populations have different modes of reproduction (sexual or asexual) which is affected by current velocity in river systems [8]. In the study of four *Ruppia cirrhosa* populations, 12 polymorphic markers (including 10 microsatellite loci and two more by cross amplification with those from *R. maritima*) were developed to reveal population diversity and differentiation [9]. In addition, the findings of genetic diversity based on SSR analysis may contribute to establishing conservation programs for some endangered species. For instance, 10 microsatellite markers have been used to assess genetic diversity of *Euryale ferox* (Nymphaeaceae), a “vulnerable” species in Japan [10]. Uesugi et al. developed 10 microsatellite markers for *Nymphoides peltata*, another threatened clonal aquatic plant, which allowed evaluation of genetic diversity and conservation design in Japan [11] and assessment of genetic diversity within and between populations in China [12].

AFLP markers combine the characteristics of RFLP and RAPD and are another effective tool to investigate genetic diversity. AFLP analysis of 30 populations of *Utricularia australis* f. *tenuicaulis* in Japan showed extremely low genetic diversity within populations in contrast to any other clonal plants. However, many of the investigated populations had highly variable and different genotypes. Character compatibility analysis explained the origin of new genotypes: rare-to-sporadic sexual reproduction, instead of somatic mutations, generated new genotypes [13].

ITS (internal transcribed spacer), ISSR, EST- (expressed sequence tag-) SSR, and other molecular markers have been developed for the analysis of aquatic plant genetic diversity. Analysis of the ISSR markers in *Ranunculus nipponicus* suggested high genetic differentiation among populations and low genetic diversity within them [14]. Due to the availability of large EST data, EST-SSR, which refers to SSR markers derived from ESTs, has been developed and used in different species. EST-SSR has higher versatility among different species and can be used for comparative genomics studies. Yuan et al. developed EST-SSR loci from an EST dataset generated by next generation sequencing in *Nymphoides peltata* to analyze and evaluate genetic diversity and structure [15].

To enhance the effectiveness of molecular markers, two molecular markers used in combination have achieved good results. The analysis of an invasive and weedy species in China, *Alternanthera philoxeroides* (Mart.) Grisb, showed that combined RAPD and ISSR could effectively identify the genetic diversity within populations [16]. These two molecular markers were also used to evaluate the genetic variability of different germplasms of an aquatic food plant, *Euryale ferox* [17].

### 2.2. Phylogenetic Analysis

High variability of aquatic plants and complexity of traits and morphology cause difficulties for phylogenetic studies. However, the DNA-based molecular marker technique has obvious advantages in overcoming the morphological limitations and determining the evolutionary relationship between different species.

*Cabomba* Aubl. is a small genus in the family Cabombaceae, containing 5 species whose identification is problematic due to vegetative similarity among the taxa. Thus in 2015, 13 SSR loci were developed to investigate the genetic structure of *C. aquatica* and assess the validity of the five recognized species [18]. Another example is the sacred lotus (*Nelumbo nucifera*), economically and ornamentally important in China. Previous classification of the Chinese lotus species relied on plant size, petal color, petal patterning, and other morphological traits. In 2011, dendrograms were constructed by AFLP or 20 novel SSR markers to study genetic relationships among 58 accessions of *N. nucifera* [19].

Three genomes exist in plant cells: nuclear, chloroplast, and mitochondrial. Due to the differences in structure and function, their evolutionary rate is different, providing alternative traits for phylogenetic studies. To reconstruct the phylogeny of the cosmopolitan aquatic plant family Hydrocharitaceae, DNA sequences from 17 genera were sampled, including eight genes: 18S from the nucleus; rbcL, matK, trnK5' intron, rpoB, and rpoC1 from chloroplasts; and cob and atp1 from mitochondria. The phylogeny showed that the Hydrocharitaceae originated in oriental area and dispersal has been the major factor forming the current transoceanic distribution of Hydrocharitaceae [20]. Moreover, ancestral state reconstruction of gender and leaf morphology provided valuable information for understanding adaptive evolution and leaf phenotype in aquatic monocots.

DNA sequence data from nuclear ribosomal and plastid regions has also been used and confirmed to be more efficient to study phylogenetic relatedness and phenotypic plasticity in aquatic plants. In the study of *Veronica* sect. *Beccabunga*, a phylogenetic framework for the group was developed based on these two highly variable molecular markers [21].

### 2.3. Germplasm Identification

The identification of aquatic plant species is difficult because of interspecific hybridization and cryptic species that are reproductively isolated but have no distinguishing morphological criteria. The morphological constraints can be overcome by molecular markers which detect differences between aquatic plant species objectively and accurately at the level of DNA.

The pondweed genus *Potamogeton* (Potamogetonaceae) has been known for its ability to hybridize extensively and prevalence of cryptic species. Identification of hybrids in linear-leaved *Potamogeton* species is difficult due to its inadequate and obscure morphological differences. The origin of *Potamogeton ×maëmetsiae*, as a new hybrid between two linear-leaved species, *P. friesii* and *P. rutilus,* was determined by AFLP analysis, nuclear (ITS, 5S-NTS (nontranscribed spacer)), and chloroplast (rpl32-trnL intergenic spacer) DNA sequence data [22].

Another species of *Potamogeton*, *P. clystocarpus*, is considered an endangered aquatic plant in Texas, but its taxonomic status was uncertain due to the lack of fixed morphological differences between it and two sympatric congeners, *P. pusillus* and *P. foliosus*. The genetic uniqueness of *P. clystocarpus* was confirmed using AFLP markers in combination with sequences of the internal transcribed spacer (ITS) region and the chloroplast *trnL-F* intron and spacer [23].

## 3. Comparative Genomics

Comparative genomics is an approach to compare the known structure of genes and genomes based on genome mapping and sequencing. Through comparing genomic sequences between different species, we can identify the coding and noncoding regulatory sequences and sequences unique to a given species. Via genome-wide sequence alignment, the similarities and differences of nucleotide composition, collinearity relationships, and the order of genes between different species can be understood, and it is conducive to predict genetic analysis and uncover biological evolutionary relationships.

In the study of the bladderwort plant *Utricularia gibba*, the coding sequences were compared to those in the terrestrial species Arabidopsis, grape, tomato, *Mimulus*, and papaya genomes using two approaches. First, comparison of Pfam domains and examination of significant differences by LRT (likelihood ratio test) showed no significant differences for most domain groups (97%). For a more in-depth study of specific differences in the genetic repertoire of *U. gibba*, gene families were classified. Among the total 18,991 gene families, 1275 have no *U. gibba* members, while 1804 showed expansions in *U. gibba* compared to others, and some gene families are specifically reduced or lost in *U. gibba* [24]. Furthermore, these gene families were classified and related to main phenotypic features. Together, the analyses suggest numerous key genes and gene families for further functional confirmation and adaptive roles in *U. gibba*'s unique lifestyle and highly specialized body plan [25].

The genome of another aquatic plant *Spirodela* exhibits a reduced gene number, but it still has representatives of 8255 gene families common to *Arabidopsis*, tomato, banana, and rice. Some genes are lost, and the copy number of gene families varies, likely consistent with its floating characteristics, compact morphogenesis, and suppression of juvenile-to-adult transition [26].

Apart from the above cases, expressed sequence tags (ESTs) have been applied to comparative genomics. Over 12,000 ESTs from the diatom *Phaeodactylum tricornutum* have been generated, and 5108 sequences have been obtained through assembly. The sequences were compared with those of other eukaryotic algae including the red alga *Cyanidioschyzon merolae* [27], the green alga *Chlamydomonas reinhardtii* [28], and the centric diatom *Thalassiosira pseudonana* [29], whose genomes are available. Through the comparison, differences between the two major diatoms were identified and genes were found encoding ACLs (ATP-citrate lyases), CAs (carbonic anhydrases), and FBAs (Fru-1,6-bisphosphate aldolases), related to general cell metabolism [30].

## 4. Functional Genomics

It has been known from comparative genomics that the relationship between genes can be explained by homologous families. In general, the homologs maintain the same or similar functions during evolution. Thus, the availability of comparative genomics strategies and molecular maps for aquatic plants has facilitated studies on functional genomics.

### 4.1. Photosynthesis-Related Genes

The unique living environment of aquatic plants has led to considerable interest in their photosynthesis, with several studies in recent years being done at the molecular level.

The aquatic monocot *Hydrilla verticillata* is a C4 NADP-malic enzyme species in which a facultative C4 cycle coexists with the C3 cycle (Calvin cycle) in the same cell. A functional genomic approach to identify elements necessary for the C4 system in *H. verticillata* used differential display (DD-RT PCR). The study identified 13 genes upregulated or uniquely expressed in C4 leaves by macroarray analysis, northern and semiquantitative RT-PCR analysis [31]. Among these, one gene (*hvpepc4*) encoded the C4 photosynthetic PEPC (phosphoenolpyruvate carboxylase) (which was substantially upregulated especially in the light), two encoded distinct isoforms of PPDK (pyruvate orthophosphate dikinase), and genes encoding an aminotransferase, a transporter, and two chaperonins were also upregulated.

The enzyme Rubisco (ribulose-l,5-bisphosphate carboxylase/oxygenase) assimilates carbon dioxide during photosynthesis and is the most abundant chloroplast enzyme in plants. The genes (*rbcS*) encoding the small subunit (SSU) of Rubisco comprise a small family in the nucleus, of which six *rbcS* genes from *L. gibba* genomic libraries were isolated in 1990 [32]. Using specific probes from the 3′-UTR of these genes, SSUl was found to be highly expressed in both roots and fronds, whereas SSU5B was expressed at extremely low level in steady-state root mRNA. The localization of these two gene transcripts was the same in fronds by in situ hybridization, indicating that the expression difference of individual *rbcS* genes in *Lemna* between organs may be determined by an organ-specific mechanism that involves posttranscriptional events [33].

Rubisco activase (RCA) is a key regulatory element in photosynthesis in aquatic plants. RACE amplification of two full-length cDNAs encoding RCA (SGrca1 and SGrca2) from the aquatic plant *Sagittaria graminea* revealed novel alternative splicing of RCA [34]. The analysis of RCA gene expression pattern demonstrates that the aerial and submerged environments regulate RCA gene expression at both transcriptional and posttranscriptional levels.

### 4.2. Stress-Related Genes

Concerns are mounting about abiotic stresses (e.g., heat, environmental pollution, and heavy metals) in the aquatic environment that affect plant growth. Studying stress-related genes in aquatic plants is of great value in fostering new resistant varieties which can be used for environmental remediation and to monitor environmental pollution.

The HSR (heat shock response) is a regulated response involving many elements [35, 36]. Comparative studies of two *Potamogeton* species revealed that heat acclimation leads to species-specific differences in heat response. The study also identified *HSFA2* (heat shock transcription factor A2) and its putative target gene *CP-sHSP* (chloroplast-localized small heat shock protein) and found that the more heat tolerant species maintained a higher transcriptional level of duplicated *HSFA2* and *CP-sHSP* genes in order to overcome severe heat stress [37].

Phytoremediation is an inexpensive, effective, and ecofriendly technology that allows environmental cleanup by plants [38]. Many macrophytes are efficient in treatment of wastewater and accumulation of heavy metals such as arsenic (As) and cadmium (Cd) [39–43]. Previous studies have applied physiological, biochemical, toxicological, and cytobiological methods to reveal the responses and mechanisms of As detoxification [44–46]. To study functional genes in this process, a PCS (phytochelatin synthase) gene (*CdPCS1*) was isolated from the aquatic plant *Ceratophyllum demersum* using a homology-based PCR approach. The role of *CdPCS1* in biosynthesis of PC (phytochelatin) was confirmed by its ability to enhance Cd and As accumulation in transgenic tobacco and *Arabidopsis* plants [47]. It was also found that the expression of *CdPCS1* in rice enhanced accumulation of As in roots, restricting accumulation in aerial parts including grain [48].

Because the ROS (reactive oxygen species) produced by the interaction between copper ions and oxygen are highly destructive, copper also has adverse effects on environment and biological systems [49]. To elucidate the molecular responses stimulated by excess copper exposure in *Lemna gibba,* genes with altered expression in the presence of copper were identified by ddPCR. Of the six genes identified, northern hybridization analysis demonstrated that four (callose synthase, HSP90, serine decarboxylase, and the biotin carboxylase subunit of acetyl-coenzyme A carboxylase) increased in expression, while the other two genes (the HAP5 subunit of the heme-activated protein (HAP) transcription factor and the chloroplast nucleoid DNA-binding protein CND41) decreased in expression. Interestingly, the altered expression corresponded to the known mechanism of copper toxicity, enabling their use as biomarkers for copper and other environmental pollutants [50].

How to enhance nutrient utilization efficiency of crops has been the subject of intensive research in recent years. To understand the mechanism of high efficiency nutrient acquisition and utilization, the genes involved in low-sulfur tolerance were isolated from a water hyacinth, *Eichhornia crassipes*, by a gene mining method. Genes were then overexpressed in Arabidopsis wild-type plants to screen for mutants. Overexpression of the jacalin-related lectin gene (*EcJRL-1*) resulted in Arabidopsis plants with improved sulfur tolerance, and a role in root elongation under sulfur-deficient conditions was confirmed [51].

## 5. Large-Scale Genome/Transcriptome Sequencing and Its Utilization

The functional genes discussed above were obtained by automated sequencing for bioinformatics analysis and preliminary verification of functions. Up to now, the genome size of many aquatic plants has been determined using flow cytometry (FCM) [52]. This genome size information will lay the foundation for future work in genome sequencing and finding a suitable model plant.

During the past two decades, based on the availability of complete sequences of chloroplast genomes, the chloroplast genome and its evolution have been studied by molecular methods. Among aquatic plants, the sequences of chloroplast genome have been accomplished for *Nuphar advena* [53], *Najas flexilis* [54], *Elodea canadensis* [55], *Utricularia foliosa* [56], *Lemna minor* [57], and three other species in different genera of the Lemnoideae—*Spirodela polyrhiza*, *Wolffiella lingulata*, and *Wolffia australiana* [58]. The sequencing technique was also upgraded from the first generation sequencer ABI 3100 and ABI 3730 to high-throughput next-generation sequencing (NGS) technologies, like Illumina, Roche/454, and AB SOLiD [59].

In contrast to chloroplast genomes, the number of sequenced mitochondrial genomes in plants is limited due to their unstable and complex structure. The mitochondrial genome of *Spirodela polyrhiza* has been sequenced from total genomic DNA [60]. In 2013, the complete nucleotide sequence of mitochondrial for *Butomus umbellatus* was described and compared to the sequenced angiosperm mitochondrial genomes [61].

Little has been reported about whole genome sequencing (WGS) of aquatic plants. *Spirodela polyrhiza*, which has the smallest monocot genome to date with the size of 158 Mb, was the first genome to be fully sequenced [62]. The genome of sacred lotus (*Nelumbo nucifera* Gaertn.) was sequenced in 2013 [63]. In 2015, the first draft genome of the aquatic model plant *Lemna minor*, another genus in the Lemnaceae, became available and will be widely utilized for future biotechnological application and stress physiology research [64].

Next-generation sequencing technologies make it possible to sequence the transcriptomes of nonmodel plants to an unprecedented extent. For example, the transcriptome of *Utricularia vulgaris* was sequenced by 454 pyrosequencing and compared to the transcriptome of *U. gibba* previously published in 2011 [65, 66]. Additionally, the transcriptome of the submerged aquatic plant *Ranunculus bungei* has been isolated and sequenced to help us understand the molecular adaptive mechanism to aquatic habitats [67]. The study of some aquatic plants with known medicinal properties is limited due to insufficient genomic and physiological information. Currently, comprehensive analysis of the transcriptome of *Nasturtium officinale* and *Oenanthe javanica* has been performed to annotate functional genes, promoting the studies of medicinal properties and corresponding pathways [68, 69].

In addition to the rise of genome sequencing, there has also been focus on noncoding RNA (ncRNA). These RNAs are involved in physiological activities across all levels in cells. In 2013, the identification of 81 conserved microRNAs (miRNAs) grouped to 41 miRNA families and 52 novel miRNAs (49 families) was reported in sacred lotus by sequencing small RNA libraries. These miRNAs were predicted to regulate 137 genes related to growth and development and other biological processes in sacred lotus [70]. In 2015, computational approaches were used in miRNA identification in sacred lotus and found 106 conserved miRNAs [71]. By combining experimental and bioinformatic analysis, these miRNAs can be used for further research of their roles in *N. nucifera*.

Though many other types of ncRNAs such as transfer RNA (tRNA), ribosomal RNA (rRNA) and small interfering RNAs (siRNAs) have not yet been reported in aquatic plants, and this “RNA world” will become a focus of attention.

## 6. Future Prospects

Compared with terrestrial plants, the number of aquatic plants is relatively few, but they provide people with food, vegetables, pharmaceuticals, fiber materials, and livestock feed. Also, they possess great value to ecology and landscape. Owing to these advantages, basic research involving aquatic plants is becoming increasingly emphasized worldwide. Through progress in aquatic plant genomics, advances have been made in terms of molecular marker development and application, isolation, and sequencing of functional genes, as well as in comparative genomics (Table 1). However, the particularities of aquatic plants have resulted in many deficiencies in understanding, thus lagging behind other model plants and crops, so there is still much to be done in aquatic plant genomics research.

With the rise of massive sequencing technologies, sequence read lengths increase and sequencing cost declines. Now, the third-generation sequencing (TGS) or “next-next” generation sequencing technologies such as the Single-Molecule Real-Time (SMRT™) Sequencing, Heliscope™ Single Molecule Sequencing, and the Ion Personal Genome Machine™ are available, which generate longer reads in a faster time with higher accuracy [72]. These newly emerging technologies will have potential for extensive application in aquatic plant genomics research.

In the future, a growing number of aquatic plant whole genomes will be sequenced, which will allow emphasis on gene functional studies. Genome editing, using “molecular scissors” or engineered nucleases, is an effective technology to target DNA or edit gene at specific sites in the genome. Currently, four types of engineered nucleases are used for genome editing: zinc finger nucleases (ZFNs) [73], engineered meganucleases, transcription activator-like effector nucleases (TALENs) [74–76], and CRISPR (clustered regularly interspaced short palindromic repeats)/Cas9 (CRISPR-associated) system [77]. Among these four tools, TALEN and CRISPR/Cas9 are used more widely in organisms, particularly the CRISPR/Cas9 system owing to its easier and faster operation. TALEN technology has been used to engineer plant crops such as rice, barley, and maize [78–80], and since 2013, CRISPR/Cas9 has been broadly applied to plants including Arabidopsis [81, 82], tobacco [82, 83], sorghum, rice [81, 84, 85], wheat [86], and maize [85]. These technologies can soon be applied to aquatic plant research, allowing identification of genes regulating important traits and construction of abundant mutants by genome editing methods.

In conclusion, to achieve comprehensive development of aquatic plant genomics, we still need to do research into these areas: (i) establishment and optimization of genetic transformation systems; (ii) whole genome deep sequencing and comparative genomics; (iii) transformation of the large fragment gene and functional genomics research; (iv) development and application of various molecular markers; and (v) construction of integrated databases, combining information on genome and proteome sequences, mRNA and protein expression profiles, and other useful information. Indeed, while genomics will facilitate the in-depth study of individual genes, proteins, or biological processes in aquatic plants, it cannot be used as a stand-alone tool. Rather, it should be integrated with other disciplines such as molecular biology, genetics, bioinformatics, biochemistry, and physiology, which will rapidly promote the study and understanding of aquatic plants.

## Figures and Tables

**Table 1:** Summary of the genomics studies and technologies correlated to aquatic plant species discussed in the review.

Genomics studies	Species	Applications
*Molecular markers*		
SSR	*Sparganium emersum* [8], *Ruppia cirrhosa* [9], *Euryale ferox* [10], *Nymphoides peltata* [11, 12]	Genetic diversity analysis
	*Cabomba aquatic* [18], *Nelumbon ucifera* [19]	Phylogenetic analysis
AFLP	*Utricularia australis* [13]	Genetic diversity analysis
	*Nelumbon ucifera* [19]	Phylogenetic analysis
	*Potamogeton ×maëmetsiae* [22], *Potamogeton clystocarpus* [23]	Germplasm identification
ISSR	*Ranunculus nipponicus* [14], *Alternanthera philoxeroides* [16], *Euryale ferox* [17]	Genetic structure
EST-SSR	*Nymphoides peltata* [15]	Genetic diversity and structure
RAPD	*Alternanthera philoxeroides* [16], *Euryale ferox* [17]	Genetic diversity
Nuclear, chloroplast, and mitochondrial markers	Hydrocharitaceae(family) [20]	Phylogenetic analysis
	*Potamogeton ×maëmetsiae* [22]*, Potamogeton clystocarpus* [23]	Germplasm identification
Plastid markers	*Veronica* sect*. Beccabunga* [21]	Phylogenetic and taxonomic analysis

*Comparative genomics*		
Interspecies comparative genomics	*Utricularia gibba* [24, 25]	Gene family classification and functional gene prediction Adaptive molecular evolution analysis
	*Spirodela* [26]	
	*Phaeodactylum tricornutum* [30]	

*Functional genomics*		
PCR approach	*Hydrilla verticillata* [31]	Identify elements necessary for C4 system
	*Potamogeton* [37]	Identify *HSFA2* and its putative target gene *CP-sHSP*
	*Ceratophyllum demersum* [85]	Identify the role of *CdPCS1* in biosynthesis of PC
	*Lemna gibba* [50]	Elucidate the molecular responses stimulated by excess copper exposure
Specific probes from 3′-UTR	*Lemna gibba* [33]	Analyze the expression of the six *rbcS* genes in different organs
RACE	*Sagittaria graminea* [34]	Analyze two *RCA* genes(*SGrca1* and *SGrca2*) expression pattern
Gene mining method	*Eichhornia crassipes* [51]	Isolate genes involving low-sulfur tolerance to understand the mechanism of high efficiency nutrient acquisition and utilization

*Genome/transcriptome sequencing*		
Chloroplast genome	*Nuphar advena* [53], *Najas flexilis* [54], *Elodea canadensis* [55]*, Lemna minor* [57], *Utricularia foliosa* [56], *Spirodela polyrhiza*, *Wolffiella lingulata* and *Wolffia australiana* [58]	Important source of genetic markers for phylogenetic analysis Identify functional coding regions and sequence outside of coding regions Chloroplast transformation for genetic engineering Evolution of chloroplast genomes
Mitochondrial genome	*Spirodela polyrhiza* [60], *Butomus umbellatus* [61]	Study the evolution of monocot mitochondrial genomes
Whole genome	*Spirodela polyrhiza* [62]	Stimulate new insights into environmental adaptation, ecology, evolution, and plant development Future bioenergy applications
	*Nelumbo nucifera* [63]	Study the evolutionary history of the genome and genes involved in relevant processes governing the unique features
	*Lemna minor* [64]	Understand the biological and molecular mechanisms in *L. minor* Facilitate future genetically improvements and biomass production applications of duckweed species
Transcriptome	*Utricularia vulgaris* [65, 66]	Identify gene losses and duplications during the course of evolution Study adaptations related to the environment and carnivorous habit and evolutionary processes responsible for considerable genome reduction
	*Ranunculus bungei* [67]	Study the molecular adaptive mechanism from terrestrial to aquatic habitats
	*Nasturtium officinale* [68], *Oenanthe javanica* [69]	Annotate function genes Promote the studies of medicinal properties and corresponding pathways
MicroRNA	*Nelumbo nucifera* [70, 71]	Identify conserved microRNAs and their target genes
